# Development of an easy-read version of the Brain Injury associated Visual Impairment - Impact Questionnaire (BIVI-IQ)

**DOI:** 10.1177/02692155261424458

**Published:** 2026-03-05

**Authors:** Lauren R Hepworth, Claire Howard, Janet Rockliffe, Jamie J Kirkham, Gill Pearl, Fiona J Rowe

**Affiliations:** 1 Institute of Population Health, 4591 University of Liverpool, Liverpool, UK; 2 523611 Salford Royal Hospital, Northern Care Alliance NHS Foundation Trust, Salford, UK; 3 VISable, patient and public representative, Liverpool, UK; 4 Centre for Biostatistics, 5292 The University of Manchester, Manchester Academic Health Science Centre, Manchester, UK; 5 Speakeasy, Bury, UK

**Keywords:** Quality of life, visual impairment, acquired brain injury, aphasia, cognitive impairment, accessibility

## Abstract

**Objective:**

The Brain Injury associated Visual Impairment - Impact Questionnaire (BIVI-IQ) was developed to measure the impact of post-brain injury visual impairment. Communication and cognitive impairments are common brain injury sequelae and a barrier to completing standard patient reported outcome measures. The objective of this study was to develop and refine an easy-read version in order to meet recommendations to promote self-reporting.

**Design:**

Easy-read version development involved stakeholder input at several meetings. An iterative refinement process was used, involving a cognitive interview, until no new issues were identified.

**Participants:**

Stroke survivors with visual impairment and/or aphasia and relevant healthcare professional were involved in the development stage. The clinical study recruited 12 stroke survivors with visual impairment.

**Main measures:**

Participants completed the easy-read and standard versions. The researcher documented observations and time taken. Analysis assessed association and agreement between the two versions.

**Results:**

Development considered the structure, image selection and key words. Four iterations were used before five consecutive participants reported no new issues. Image amendments involved replacing four, revising four and adding three across eight of the 13 items. A preference for the easy-read version was reported by 45.5% completing both questionnaires (n = 11). A significant, strong positive correlation was found between the easy-read and standard version total scores. Weighted Kappa found significant agreement between 12 items.

**Conclusions:**

The easy-read version, using images to support the question wording, will increase accessibility for brain injury survivors with communication and/or cognitive difficulties. It is acceptable and further evaluation of this version is now required.

## Introduction

The incidence of post-stroke visual impairment is reported as 60% for stroke survivors.^1^ The prevalence of aphasia post-stroke is reported as between 30–34% in the acute stages.^2^ Cognitive difficulties following brain injury are also common with reported prevalences up to 91% with at least one cognitive domain deficit.^3–5^ Brain injury survivors with aphasia regardless of severity, often have a co-existing degree of alexia resulting in difficulty reading and comprehension of written word.^6^ Therefore, there is likely to be a high degree of crossover between communication difficulties, cognitive impairment and visual impairment.

The Brain Injury associated Visual Impairment - Impact Questionnaire or BIVI-IQ was developed to measure the impact of post-brain injury visual impairment.^7^ As with many patient reported outcome measures it was designed as a self-reporting tool, although validation used a combination of self-reporting and interview delivery.^8^

There have been several studies which have reported issues with the high literacy levels at which patient reported outcome measures are written across various disease areas.^9,10^ Other studies have identified further barriers to completion of patient reported outcome measures in addition to print literacy, including language proficiency and cognitive functioning.^11^

Patient reported outcome measures provide a vehicle for patients to inform clinicians and/or researchers of their views on the social, psychological and emotional impact of their health status.^12,13^ It is possible for several people with the same level of disability to perceive themselves as having different levels of quality of life due to their personality and what they view as important. It is this ‘subjectivity’ which is the central focus when measuring quality of life.^14^ One solution for assessing quality of life with individuals with cognitive or communication difficulties, where it can be viewed self-reporting is unreliable, is the use of a proxy.^15^ The chosen proxy is commonly someone that knows the individual well, i.e., a family caregiver. The use of proxies can be valid, especially when tools have been specifically designed for this method of delivery, however there are limitations when assessing constructs/conditions which cannot be appreciated by others e.g., visual impairment.^15–18^ Therefore, other alternatives are required to assess vision-related quality of life.

Efforts have been increasing to make health information more accessible. Various terminology is used to refer to such materials, such as ‘easy-read’ and ‘plain English’.^19^ ‘Plain English’ documents are required to use everyday words with an absence of jargon for the general population. These adaptations do not go as far as ‘easy-read’ documents which use simple words, short sentences, additional white space and pictures to support the text for individuals with a disability which impacts reading.^19^

A recent scoping review which aimed to identify easy-read adaptation of patient reported outcome measures, found 19 articles reporting adaptation of communication approaches for measuring quality of life.^20^ The majority adapted previously existing instruments including adaptation to the questions and response categories. They recommended an interdisciplinary approach to development of an easy-read version of a patient reported outcome measure, to reduce use of proxies and promote self-reporting.^20^

The aim of this study was to develop and refine an easy-read version of the Brain Injury associated Visual Impairment - Impact Questionnaire, for it to be more accessible for brain injury survivors with aphasia and cognitive impairment.

## Methods

### Initial development

The standard version Brain Injury associated Visual Impairment - Impact Questionnaire, comprises 13 questions asking about the amount of difficulty felt in relation to different daily activities and experiences, with a 4-point scale for each question presented in a table format.^7^

A pilot easy-read version was developed following stakeholder input at four meetings (face-to-face or virtual) and through offline communications (e.g., emails, telephone conversations). Meetings and conversations were not recorded but notes of key points and decisions were taken. The stakeholder input was sought from professional experts in the field, the VISable (a patient and public involvement group comprising lay people with experience of post-stroke visual impairment, facilitated by the VISION research team at the University of Liverpool) and Speakeasy group (regular patient and carer support and activity group for individuals with aphasia). The only group to be consulted more than once was the Speakeasy group, therefore it is possible individuals attended both meetings where the study was presented and discussed. All provided verbal or non-verbal indications of informed consent for their involvement. Appropriate reimbursement for time was offered to individuals not attending as a healthcare professional. Stakeholders included stroke survivors with visual impairment, stroke survivors with aphasia, speech and language therapists, occupational therapists and orthoptists. Whilst the two groups consulted were targeted at specific post-stroke sequelae, visual impairment or aphasia, the two groups were not completely distinct with some members experiencing both conditions.

No published principles or guidelines were used. Advice was sought on the following elements based on experience of a member of the research team in developing aphasia-friendly materials:

1. General layout, i.e., how many questions per page, spacing etc.,
2. Key words within the question which needed highlighting,
3. Response option appearance,
4. Pictorial representations for each question.

Once a version had been agreed, it was then taken forward for use in a clinical study for refinement.

### Refinement

This study was performed in line with the principles of the Declaration of Helsinki. Approval was granted by a NHS Research Ethics Committee (20/EE/0120).

Stroke survivors with visual impairment were recruited from a single NHS hospital, either from the stroke unit or eye clinic. Individuals over 18 years of age, with a clinical or radiological confirmed stroke with related visual impairment and capable of self-completing a questionnaire were eligible for inclusion. Those with severe communication or cognitive difficulties or unable to provide informed consent were excluded. These decisions were made following discussions with the clinical multi-disciplinary team.

Demographics, clinical details in relation to stroke and diagnosed stroke sequelae were extracted from the medical records. Visual impairment diagnosis was collected from the routine orthoptic assessment completed at the time of recruitment.

Participants were asked to complete the easy-read version of the Brain Injury associated Visual Impairment - Impact Questionnaire, followed by the standard version in paper format, during a face-to-face clinical consultation, with a comfort break in-between. A randomised order of the versions was not used as the easy-read version was the focus of the study. Time taken was measured and observational notes were taken whilst the participant completed the questionnaires. A cognitive interview followed the questionnaire completion exploring if the images were an accurate representation of the words. The interview was completed by the clinical researcher undertaking the clinical assessment and study recruitment. An interview guide was followed comprising two sections. Section one initially asked an open question about their experience completing the questionnaires followed by which of the two versions they preferred completing. Section two was repeated for each page of the questionnaire and asked about meaning of the picture, supportiveness of the picture to the wording, what the question was asking, and finally about the meaning of specific terms/phrases in the question. The findings from the questionnaire completion, observation and interview were used in an iterative process to modify the easy-read version for future participants. If participants indicated ambiguity regarding the pictures or wording within the easy-read version, these were discussed as to whether an amendment should be made and if so, what changes were required. This cycle was repeated until there were no new issues identified with question pictorial representation identified.

Descriptive statistics were used to report the sample characteristics and missing data. The scores of the easy-read and standard versions of the Brain Injury associated Visual Impairment - Impact Questionnaire completed were compared. Spearman correlation was used to quantify the association between the Brain Injury associated Visual Impairment - Impact Questionnaire converted total score completed using the easy-read version and the standard version.^8^ Weighted Kappa (K) was used to assess chance-eliminated agreement between the individual items completed using the easy-read version and the standard version.^21^

## Results

### Initial development

Version 1 of the easy-read version was developed through a process of facilitated discussion at two consultation group meetings. Visual presentation of information to the group was used to obtain feedback regarding accessibility and individuals’ preferences. Alterations were made for further discussion.

The initial meeting focused on the overall structure of the easy-read version, including selection of images and key words to be highlighted. To allow focus on each question individually, the decision was taken to have a single question to a page. Based on past research it is known images are helpful to aid comprehension of written materials. Research into preference of image style had already been conducted, which included the co-development of images with stroke survivors with aphasia.^22,23^ A suite of >200 images are publicly available, from Speakeasy (www.speakeasy-aphasia.org.uk), for use to make stroke research documentation more accessible. These images were designed in a style appropriate for adults, they have variation in skin tone, body shape and gender to be representative of the broader population and were specifically designed with detail to enhance understanding.^22^ Images that portrayed the correct meaning of each question were selected from this image bank; missing required images were noted. New images were created where needed using the same process described for the creation of the original image set.^22^ The subsequent meeting focused on the fine detail of the easy-read version including word spacing (two spaces between each word), picture location (placed after the question and before the questionnaire options) and response option appearance (using a combination of colour and size in addition to words, to indicate the positive and negative ends of the scale).

### Refinement

A total of 12 stroke survivors with post-stroke visual impairment were recruited. One participant was recruited following their vision assessment, however, did not complete any questionnaires due to fatigue and their visual impairment had recovered at the subsequent follow-up appointment.

The average age at time of recruitment was 66.5 (SD 12.4) years and six (50%) were female. One participant was diagnosed with expressive aphasia and four participants were diagnosed with cognitive impairment. The following types of visual impairment were diagnosed: visual field loss (n = 8, 66.7%), reading difficulties (n = 6, 50.0%), reduced visual acuity (n = 3, 25.0%), ocular motility defect (n = 3, 25.0%) and visual perception deficits (n = 3, 25.0%). Some participants had multiple types of visual impairment diagnosed. The median time post-stroke was 57 days (range 1 day to 15.7 months).

Four versions of the easy-read version were created iteratively and consecutively before a series of five participants reported no new issues. The following sections outline the changes made over these four versions (Table 1) with associated quotes that triggered the amendments.

**Table 1.**
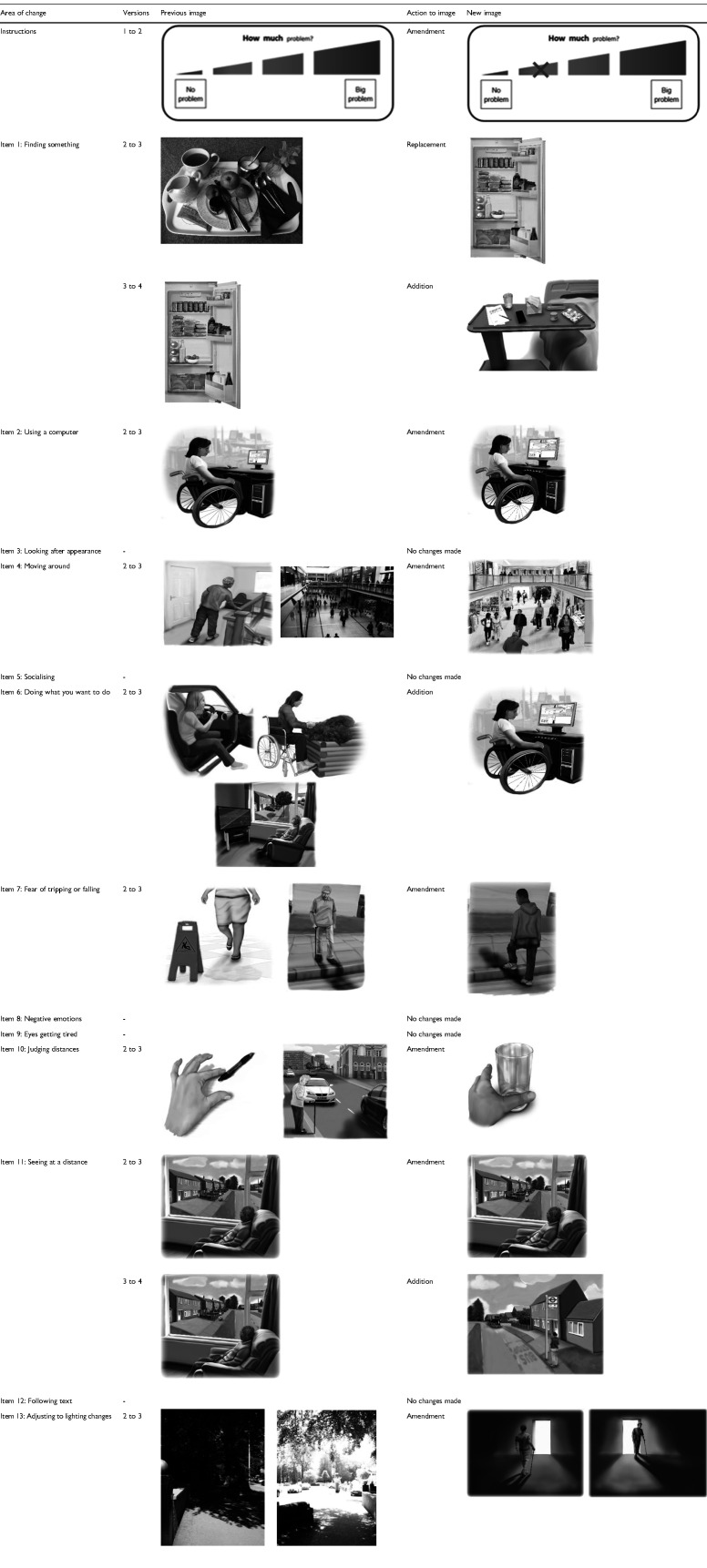
Changes made to images in the Brain Injury associated Visual Impairment - Impact Questionnaire easy-read version.

One participant contributed to the changes creating version 2. During completion of the easy-read version, and observations, the participant asked the question of where to mark their answer. In response to this an example answer was added to the instructions page.

Four participants contributed to the changes creating version 3. This amendment involved the alteration of eight pictures across seven items and the addition of one picture to one item. The picture for item 1 (finding something) had been commented on by several participants as not portraying the meaning of the question very well. The interpretations that had been made on the original picture of a busy tray were “*it's quite cluttered, it's a tea, some sort of … tea party” *[ER02]*, “it means I am getting something to eat”* [ER04]. As a result, the picture for item one was replaced by an open fridge with lots of items. It was also transformed from a photo to a pictorial format to match those in other items. For item 2 (using electronic devices), one participant reported “*one's putting me off because of the wheelchair”* [ER03]. As a result, the proportions in the picture were adjusted so the focus of the picture (which was the computer) was a more appropriate size. Item 4 (moving around) was a picture of a shopping centre and received the suggestion “*there's nothing about me being there, it's just a picture of summat, so perhaps … if you somehow got a person walking along”* [ER04]. Therefore, the photo was changed to a pictorial format to match those in other items and better portray a person being in the busy space. Item 7 (fear of tripping or falling) portrayed a person walking down a curb, after the following comment “*a raised curb something that I could actually trip on”* [ER04]. The picture was altered to show someone stepping up to the curb. One of the images for item 10 (judging distances) was a hand aiming to pick up a pen. It was suggested that a glass may be a better object to be reaching for as this would be done by everyone. This picture was changed accordingly. The picture for item 11 (seeing far away) was not interpreted correctly by more than one participant; “*he just looks a bit sad there on his own”* [ER03], *mean's stuck at home and can’t go out”* [ER04]. Therefore, an alteration to the picture was made adding a focus of something to look at in the distance. Item 13 (adjusting to different lighting) was commented on by a number of participants, interpreting the pictures as appreciation of light and dark rather than adjusting to a change in lighting; “*do you recognise light and dark”* [ER01], “*that's lighter than that one, I’d sooner be there it looks warmer”* [ER04]. A suggestion was made “*it's not like there is anyone stepping out from the dark into the light”* [ER03]. Therefore, the photo was changed to a pictorial format to match those in other items and add a person moving between the different lighting states. An additional picture of someone on a computer was added to item 6 (doing what you want to do) in response to the previous pictures only depicting older people “*Driving yeah, watching tele yeah, maybe some different activities like maybe … somebody at work”* [ER03].

Two participants contributed to the changes creating version 4. This amendment involved the addition of two pictures across two items. One participant commented that the fridge in item 1 (finding something) would not be relevant for an inpatient hospital setting. Therefore, a hospital overbed table was added with lots of items scattered across it. It was noted that item 11 (seeing far away) continued to lack clarity; “*he's being nosy … he's watching this woman coming up the road* *…* *but no he's relaxing”* [ER06], “*it's a bit sad staring out of the window all the time”* [ER07]. A suggestion was made for an alternative picture “*sort of getting bigger and bigger”* [ER06]. A previous suggestion from version 2 had also been made; “*it's like when you have your driving test and they say … what's the registration of the white car”* [ER04]. Therefore, the decision was taking to add a picture of an approaching bus.

### Comparison

The median of the difference of time taken to complete the easy-read and standard versions was 120 seconds, with a range of −111 (easy-read quicker) to 272 (standard quicker) seconds. Overall, the standard version was completed quicker in a median of 109 seconds (range 54 to 359), compared to the easy-read version completed in a median of 248 seconds (range 131 to 515).

Of the 11 participants completing both questionnaires, 45.5% (n = 5) reported preference for completion of the easy-read version. Using a Spearman's rank order correlation, there was a statistically significant, strong positive correlation between the total scores of the easy-read and standard versions of the Brain Injury associated Visual Impairment - Impact Questionnaire (r_s_(9) = 0.913, p < 0.005).

Weighted Kappa analysis found statistically significant agreement between the easy-read and standard versions for 12 of the 13 items (Table 2). The strength of agreement ranged between 0.421 (moderate agreement) on the ‘negative emotion’ item to 0.925 (almost perfect agreement) on the ‘judging distances’ item. The items either had substantial agreement or better (n = 8, 61.5%) or moderate agreement (n = 5, 38.5%).

**Table 2.** Weighed kappa for each item of the Brain Injury associated Visual Impairment - Impact Quesitonnaire assessing the agreement between the easy-read and standard versions. Interpretation of strength of agreement as per Holey et al. (2007).

Items	Kappa (95% CI)	*P*-value	Strength of agreement
1. Finding things	0.680 (0.344–1.02)	0.018*	Substantial
2. Computer	0.648 (0.367–0.930)	0.020*	Substantial
3. Looking after appearance	0.495 (0.200–0.790)	0.046*	Moderate
4. Getting about	0.800 (0.655–0.945)	0.003*	Substantial
5. Socialising	0.624 (0.357–0.890)	0.024*	Substantial
6. Doing what you want	0.503 (0.095–0.910)	0.041*	Moderate
7. Fear of falling	0.727 (0.565–0.889)	0.014*	Substantial
8. Negative emotions	0.421 (−0.071–0.913)	0.082	Moderate
9. Tired eyes	0.716 (0.541–0.890)	0.008*	Substantial
10. Judging distances	0.925 (0.771–1.079)	0.002*	Almost perfect
11. Seeing distance	0.697 (0.313–1.082)	0.012*	Substantial
12. Reading	0.570 (0.260–0.880)	0.029*	Moderate
13. Adjusting to light	0.452 (0.193–0.712)	0.011*	Moderate

*indicates a statistically significant finding at *p* < 0.05.

## Discussion

The definition of a patient-reported outcome measure is that they are a report of an individual's health condition directly from that individual without interpretation from another person.^24^ Therefore every attempt should be made to allow for self-reporting.^20^ Despite this there have been attempts to assess the use of a proxy to complete patient-reported outcome measures for health-related quality of life, whilst they have been shown they can be valid, there are limitations to physical abilities, beyond the acute post-stroke and limited responsive to change.^16,17^ However, visual impairment is often referred to as a ‘hidden’ disability therefore measures of vision-related quality of life are unlikely to be valid using a proxy.^18^ A systematic review found no vision-related quality of life measures designed for use with a proxy and suggested that any such measure would need to focus on observable behaviour rather than personal experience and psychological impact.^25^

This study aimed to develop and refine an easy-read version of the Brain Injury associated Visual Impairment - Impact Questionnaire, in order to promote self-reporting and avoid the need for proxy reporting. The purpose of this adaptation was to increase accessibility of the Brain Injury associated Visual Impairment - Impact Questionnaire to brain injury survivors with visual impairment, who also have communication and/or cognitive difficulties. The results confirmed a strong positive correlation between the two versions, with assessment on an item level showing moderate to substantial agreement. Caution should be applied when interpreting the statistical analysis presented in this study due to several methodological limitations. The iterative nature of the refinement process means that the easy-read version compared to the standard version was not consistent across all participants. There was also no randomisation of the two versions, with the easy-read version always being completed first. Therefore, there is a likelihood that this prompted participants’ responses for the standard version.

Several studies have highlighted that individuals with aphasia and/or cognitive impairment are regularly excluded from participating in research studies.^26–28^ Aphasia alone has a prevalence of one third of stroke survivors acutely.^2^ It is important that strategies are employed to enable participation within research to reduce bias and inequalities, resources related to informed consent have already been developed, the outcome measures used within studies also need to be accessible.^22,29,30^ Patient reported outcome measures to assess health-related quality of life, have been developed or adapted to be accessible for people with communication difficulties or cognitive impairment.^31–35^ The Brain Injury associated Visual Impairment - Impact Questionnaire was originally developed as an instrument to assess the impact of the wide variety of post-brain injury (with stroke aetiology emphasis) vision-related quality of life.^36,37^ The Brain Injury associated Visual Impairment - Impact Questionnaire easy-read version adds a measure of vision-related quality of life to this collection of accessible patient reported outcome measures.

The correlation and agreement results are based on a small sample size. The standard version of the Brain Injury associated Visual Impairment - Impact Questionnaire has already been tested in terms of psychometric properties, including structural properties, construct validity, reliability and responsiveness to change in a larger sample.^8^ It is expected that the easy-read adaptation should perform in a similar way, however, this cannot be assumed. The easy-read Brain Injury associated Visual Impairment - Impact Questionnaire has potential to facilitate more brain injury survivors with cognitive and/or communication difficulties to express their vision-related quality of life without having to resort to a proxy measure, whether this be in clinical practice or research studies. Therefore, it is recommended that the retention of psychometric properties be assessed with a large sample of brain-injury survivors including those with communication difficulties and/or cognitive impairment.

The easy-read version of the Brain Injury associated Visual Impairment - Impact Questionnaire is acceptable to stroke survivors with visual impairment. Despite participants not being the target population (i.e., stroke survivors with visual impairment and communication and/or cognitive difficulties), a larger proportion than expected preferred the easy-read version. This may be due to the visual impairment they were experiencing rather than communication or cognitive difficulties. Therefore, a future study is warranted to assess which population groups could benefit from using the easy-read version over the standard version beyond the intended groups. Further testing of the easy-read version is also required with its target population of stroke survivors with visual impairment and communication and/or cognitive difficulties.

**Clinical Messages**

- The Brain Injury associated Visual Impairment – Impact Questionnaire (BIVI-IQ) is a valid measure, using the easy read version it would be possible to attain self-reported impact to vision-related quality of life from brain injury survivors with communication and/or cognitive impairment.
- The easy-read version correlates with the standard version of the Brain Injury associated Visual Impairment – Impact Questionnaire (BIVI-IQ).
